# Epitope Selection for Fighting Visceral Leishmaniosis: Not All Peptides Function the Same Way

**DOI:** 10.3390/vaccines8030352

**Published:** 2020-07-01

**Authors:** Abel Martínez-Rodrigo, Alicia Mas, Daniel Álvarez-Campos, José A. Orden, Gustavo Domínguez-Bernal, Javier Carrión

**Affiliations:** INMIVET, Departamento de Sanidad Animal, Facultad de Veterinaria, Universidad Complutense Madrid, 28040 Madrid, Spain; abelma01@ucm.es (A.M.-R.); alimas@ucm.es (A.M.); danalv05@ucm.es (D.Á.-C.); jaorden@ucm.es (J.A.O.); javier.carrion@ucm.es (J.C.)

**Keywords:** *Leishmania infantum*, in silico analysis, epitope prediction, multiepitope-based vaccine, leishmaniosis, HisDTC

## Abstract

Visceral leishmaniosis (VL) caused by *Leishmania infantum* is a disease with an increasing prevalence worldwide. Treatments are expensive, toxic, and ineffective. Therefore, vaccination seems to be a promising approach to control VL. Peptide-based vaccination is a useful method due to its stability, absence of local side effects, and ease of scaling up. In this context, bioinformatics seems to facilitate the use of peptides, as this analysis can predict high binding affinity epitopes to MHC class I and II molecules of different species. We have recently reported the use of HisAK70 DNA immunization in mice to induce a resistant phenotype against *L. major*, *L. infantum*, and *L. amazonensis* infections. In the present study, we used bioinformatics tools to select promising multiepitope peptides (HisDTC and AK) from the polyprotein encoded in the HisAK70 DNA to evaluate their immunogenicity in the murine model of VL by *L. infantum*. Our results revealed that both multiepitope peptides were able to induce the control of VL in mice. Furthermore, HisDTC was able to induce a better cell-mediated immune response in terms of reduced parasite burden, protective cytokine profile, leishmanicidal enzyme modulation, and specific IgG2a isotype production in immunized mice, before and after infectious challenge. Overall, this study indicates that the HisDTC chimera may be considered a satisfactory tool to control VL because it is able to activate a potent CD4^+^ and CD8^+^ T-cell protective immune responses.

## 1. Introduction

*Leishmania infantum* is the main causative agent of visceral leishmaniosis (VL), the most fatal form of leishmaniosis in humans. This zoonosis is considered endemic in distinct areas of the tropics, the subtropics, and the Mediterranean basin [1]. Also known as kala-azar, this disease is fatal if left untreated in over 95% of cases. The disease is characterized by irregular bouts of fever, weight reduction, hepatosplenomegaly, and anemia. An estimated 50,000 to 90,000 new cases of VL occur worldwide each year [2]. In Europe, VL is a vector-borne disease mainly transmitted when female phlebotomine sandflies species *Phlebotomus perniciosus* inoculate, during a blood meal, infective promastigotes into the dermis [3]. The latest human leishmaniosis outbreak in Madrid (Spain) reinforced the endemic nature and the high prevalence of the disease in the Mediterranean area [4,5,6]. Canids are traditionally considered the main domestic reservoirs for *L. infantum*. Nevertheless, hares and rabbits of the focus area of Madrid have been the main relevant competent reservoirs in the largest reported community outbreak of human leishmaniosis in Europe [7,8,9].

Despite being an old known disease [10], leishmaniosis is still among the three major neglected tropical diseases (NTDs) caused by protozoa [11], being the second major cause of mortality among tropical infections [12]. In addition, a large number of studies on leishmaniosis have been recently reported, showing that the parasites are spreading into new geographical areas worldwide by adapting to changing environments [5,13,14].

In this context, immunization seems to be a better approach to control VL as current treatments are highly toxic, highly expensive, of extended duration, and prone to increasing parasite drug resistance [15]. Interestingly, while subjects who recovered from the disease may develop immunity and become resistant to subsequent infections, a successful immunization should be able to induce specific memory T-cells that can be maintained over time [16]. An effective vaccine must also promote the development of antileishmanial CD8^+^ and CD4^+^ Th1 cell-mediated immunity, which is mainly characterized by the synthesis of interferon-γ (IFN-γ) and IL-12, along with other proinflammatory cytokines [17].

Despite extensive research in the field of advancement towards vaccine development and enhanced understanding of VL immunopathogenesis, there is no vaccine licensed for immunoprophylaxis or immunotherapy against human VL [15]. Although there are currently three vaccines that are commercially available for immunization against canine leishmaniosis (CanL) (LetiFend^®^, Leish-Tec^®^, and CaniLeish^®^), after years of marketing, there are still doubts about the efficacy and effectiveness of the vaccine, potential infectiousness of immunized and naturally infected animals, or potential issues in *L. infantum* serological diagnosis due to vaccine-induced immunoglobulins [18]. Therefore, the need to develop a vaccine for human VL and to improve existing CanL is still desirable.

Numerous studies have focused on second-generation vaccines, including recombinant proteins [19]. Recombinant proteins or defined peptides, which were in decline until several years ago, appear to be one of the most encouraging advances for the rational design of vaccination strategies. In fact, the current use of peptides for the activation of T-cells is being used for the development of vaccines against leishmaniosis [19]. Moreover, peptides can be used alone [20], in combination with some adjuvants [21], or carried by different delivery systems [22,23].

To that end, multiple studies have been conducted using immunoinformatics as a tool to predict appropriate T-cell-specific epitopes from several proteins [24,25]. Thus, the fact that T-lymphocytes from genetically distinct populations recognize and respond to a single peptide epitope underlines the need to identify epitope(s) that may bind to different MHC alleles and cover close to 100% of the genetically diverse mammalian host population [26]. Such algorithms predict promiscuous epitopes presented by different MHC supertypes, providing a way to overcome the obstacle of MHC heterogeneity in mammalian populations through the design of “polytope vaccines” against pathogens [23].

There are several groups demonstrating that various leishmanial antigens elicit desired Th1 and CTL responses capable of sustaining resistance against different experimental challenges [15]. Among these antigens, our group has previously described the polyprotein HisAK70, which is composed of four histones (H2A, H2B, H3, and H4) [27,28], the amastigote-specific molecule (A2) [29], the kinetoplastid membrane protein-11 (KMP-11) [30], and the heat shock protein Hsp70 [31], which induced a desired resistance immune-phenotype in mice against *L. infantum*, *L. amazonensis* and *L. major* infections [32,33], as well as in the CanL model [34].

In the present study, we propose a new immunization strategy using the immunogenic potential of HisAK70 cocktail peptides through the selection of strongly binding MHC class I and II-restricted epitopes for humans, mice, and other mammals. We explored the immunogenicity and immunoprophylactic use of different formulations of two rationally designed synthetic multiepitope sequences (HisDTC and AK) in combination with saponin as an adjuvant using a previously well-established experimental murine model of VL.

## 2. Materials and Methods

### 2.1. Animals, Parasites, and Production of Leishmania Soluble Antigen

Eight-week-old female BALB/c mice (Janvier-Labs, Laval, France) were maintained under specific-pathogen-free conditions according to the procedures of the UCM Animal Facilities. The study was approved by the Animal Welfare Committee of the Community of Madrid, Spain, (reference: PROEX 211/18) following Spanish and EU legislation (Law 32/2007, R.D. 53/2013, and Council Directive 2010/63/EU).

*L. infantum* (MCAN/ES/96/BCN150 zymodeme MON-1) was cultured as previously described [35] at 26 °C in Schneider’s medium (Sigma-Aldrich, Saint Louis, MO, USA) supplemented with 20% inactivated fetal bovine serum (FBS, Gibco, Life Technologies, Thermo Fisher Scientific, Waltham, MA, USA), 200 U/mL penicillin, and 200 µg/mL streptomycin (Lonza, Basel, Switzerland). The soluble *L. infantum* antigen (SLA) was obtained from metacyclic promastigotes, as stated elsewhere [36].

### 2.2. Prediction of MHC Class I and II Binding Epitopes

Full sequences of the highly immunogenic antigens (protein name, GenBank accession number) H2A (histone H2A, XP_003392461), H2B (histone H2B, XP_001263598), H3 (histone H3, XP_001463740), H4 (histone H4, XP_001464339), A2 (A2 protein, XP_001465588), and KMP-11 (kinetoplastid membrane protein, XP_001469032) of *Leishmania infantum* were obtained from GenBank data on the JPCM5 strain (MCAN/ES/98/LLm-887) and subsequently evaluated for the prediction of MHC class I and II binding epitopes. The six *L. infantum* proteins were subjected to an in silico analysis using two available online programs: NetMHC (4.0 version) (DTU Health Tech, University of Denmark, Lyngby, Denmark) for binding peptides to MHC class I molecules [37,38] and NetMHCII (2.3 version) for binding peptides to MHC class II molecules [39]. Candidate binders were selected based on the percentage (%) rank as recommended by each software. Strong binding epitopes were first selected when the rank was below 2% for NetMHC and 10% for NetMHCII.

### 2.3. Multiepitope Peptide Design and Synthesis

Based on the prediction results of the algorithms employed, 9-mer epitopes MHC class I- and 15-mer epitopes MHC class II-restricted, with high scores against HLA (A, B, C, E) and HLA (DR, DP, DQ) alleles, respectively, were selected for each *L. infantum* protein. Subsequently, the proteins were also analyzed for mouse alleles H-2 for both MHC class I and II molecules. Thus, 6 peptides, measuring 15–30 amino acids (aa) in length, were designed in a way that each peptide included one MHC class I-restricted epitope that scored very high for at least three loci, as well as at least one adjacent or overlapping MHC class II-restricted epitope that also scored high for more than one human and murine loci (Table 1). Sequence homology between each multiepitope peptide to the human and mouse proteome was analyzed on the BLAST database. Peptides with no coverage identity to the above-mentioned proteomes were selected to avoid potential autoimmunity [24]. Thus, 2 multiepitope chimeric peptides were designed as follows: HisDTC, including selected epitopes from the proteins H2A, H2B, H3, and H4, and AK with strong binding epitopes of KMP-11 and A2 protein (Table 1). The conservation of potential epitopes within these sequences was confirmed by using the same programs used to predict MHC class I and II epitopes. Both sequences were also submitted to NetTepi (1.0 version) (DTU Health Tech, University of Denmark, Lyngby, Denmark ), which predicts T-cell epitopes from protein sequences based on calculated binding affinity, stability, and T-cell propensity to 13 different human MHC alleles, representing 11 of the common HLA-A and B supertypes [40]. Furthermore, we determined the existence of high-affinity epitopes to other species available on the NetMHC server, such as chimpanzees, macaques, cattle, and pigs.

The two multiepitope chimeric peptides (HisDTC and AK) were synthesized by GenScript Biotech (Leiden, Netherlands) with purity ≥ 95%. Synthetic multiepitope peptides were dissolved in Dulbecco’s phosphate-buffered saline (PBS; Sigma-Aldrich, Saint Louis, MO, USA) according to their hydrophobicity. Peptides were stored at −20 °C until use.

### 2.4. Immunization Protocol

Immunizations were carried out in five groups of mice (*n* = 9/group) as follows: His, AK, His-AK, Saponin, and Saline groups. The animals were immunized by subcutaneous route of administration (s.c.) with 50 µg of HisDTC peptide and 25 µg of saponin for the His group; 50 µg of AK peptide and 25 µg of saponin for the AK group; 25 µg of the HisDTC peptide, 25 µg of AK peptide, and 25 µg of saponin for the His-AK group; 25 µg of saponin for Saponin group. The PBS group was inoculated with PBS by the same procedure. Peptides and saponin were previously diluted in PBS, and animals were s.c. immunized in their footpad with a total volume of 50 µL on days −60, −45, and −30.

### 2.5. Maturation of Bone-Marrow-Derived Dendritic Cells (BMDCs) for Post- and Preinfection Assays

Bone marrow stem cell progenitors were isolated from the femurs of BALB/c mice (*n* = 3) and maintained in complete medium (CM), as previously described [41]. Fresh CM containing 20 ng/mL of murine granulocyte-macrophage colony-stimulating factor (GM-CSF; PeproTech, London, UK) was added to the cultures every 3 days, and on day 7, the nonadherent cells were collected and considered BMDCs based on CD11c expression levels as described [42].

### 2.6. Immune Response Induced by Multiepitope Peptide Immunizations Prior to Infection

With the aim of analyzing the cytokine levels induced by immunization before in vivo challenge, naïve BMDCs were seeded (5 × 10^5^ cells/mL) into 24-well plates and pulsed overnight with 25 µg/mL SLA for all the groups. In parallel, 15 µg/mL HisDTC for the His group, 15 µg/mL AK for the AK group, and 7.5 µg/mL HisDTC and 7.5 µg/mL AK for His-AK and both control groups were added for restimulation. Subsequently, BMDCs were cultured together with splenocytes isolated from immunized and sacrificed mice (*n* = 4 per group) at a 1:5 ratio (BMDCs:splenocytes) as previously described [33]. After 4 days of coculture, supernatants were collected and the levels of IFN-γ, IL-12 (p40), IL-10, and IL-4 were determined by using commercial ELISA kits following the manufacturer’s instructions as follows: for IFN-γ, the Eli-pair kit (Diaclone, Besanços, France); for IL-12 (p40) and IL-10, the BD OptEIA kit (Bioscience, San Diego, CA, USA); for IL-4, the Duoset ELISA kit (Development System R&D, Abingdon, UK).

The immune resistance phenotype induced before the in vivo challenge was analyzed by measuring the ability of lymphocytes from immunized animals to confer killing activity to naïve BMDCs following the procedures as previously described [43]. A total of 400 cells per animal were counted by optical microscopy (Olympus BX41, Olympus, Tokio, Japan). The percentage of infected cells and the mean number of intracellular amastigotes per infected cell were evaluated. The infection index (percentage of infected cells × number of parasites per infected cells) was also determined to account for the overall parasite load [43].

### 2.7. Infection and Parasite Burden

To analyze the efficacy of the multiepitope peptides against *L. infantum* infection, 30 days after the last immunization, mice (*n* = 5/group) were intravenously injected with 5 × 10^5^ *L. infantum* stationary promastigotes. Six weeks postinfection, animals were sacrificed. Then, sera, spleen, and liver were collected for parasitological and immunological assessments. To analyze the parasite burden, spleen and liver were subjected to a limiting dilution assay as described elsewhere [44]. The number of viable parasites was determined from the highest dilution at which promastigotes could be observed after 10 days of incubation at 26 °C. Values are expressed per the whole organ calculated from the reciprocal of the highest dilution containing viable parasites.

### 2.8. Cellular Immune Response in the Spleen: Cytokine Production

To analyze the immunological response of immunized animals against *L. infantum*, six weeks after *L. infantum* inoculation, a coculture system consisted of splenocytes from challenged mice and naïve BMDCs was made to determine cytokine production in the supernatants, as described in Section 2.6. In parallel, the cellular immune response elicited was also evaluated by flow cytometry in the UCM Flow Cytometry Core Facility. For this purpose, spleen cells (5 × 10^6^ cells) from immunized and infected animals were collected and stimulated in vitro in polypropylene tubes (Falcon, BD Pharmingen) with SLA (25 μg/mL) for 48 h at 37 °C in 5% CO_2_, whereas the nonstimulated culture only received medium. The intracytoplasmic IFN-γ (Clone XMG1.2, Biolegend, San Diego, CA, USA) and IL-10 (Clone JES5-16E3, Biolegend, San Diego, CA, USA) producing T-cell profiles was measured (as described elsewhere [45]) in both CD4^+^ cells (Clone GK1.5, Biolegend, San Diego, CA, USA) and CD8^+^ cells (Clone 53-6.7, Biolegend, San Diego, CA, USA). All the measurements were carried out on a FACSCalibur^®^ flow cytometer (Becton Dickinson, USA), and the Cell-Quest™ software package (Franklin Lakes, NJ, USA) was used for analysis based on a total number of 50,000 events per sample. The percentage of specific cytokine-producing CD4^+^ or CD8^+^ T-cells relative to the total number of CD4^+^ or CD8^+^ T-lymphocytes was determined by analysis of FACS data using the FlowJo software package (Tree Star, Inc., Ashland, OR, USA). The results were shown as indexes calculated by the ratio of the percentage of CD4^+^ and CD8^+^ T-lymphocytes in the SLA-stimulated cultures to the values achieved for the nonstimulated cells (ratio: stimulated culture/nonstimulated culture).

### 2.9. Enzyme Modulation after Infection

The concentration of nitrites, which are a byproduct of nitric oxide (NO) production, was determined in the supernatant from the coculture system (splenocytes: SLA-pulsed BMDCs) after 4 days using the Griess reaction, as described elsewhere [46]. Subsequently, intracellular arginase activity was measured in the same cells after incubation for 30 min in lysis buffer (0.1 M Tris–HCl, pH 7.5, 300 μM NaCl, 1 μM PMSF, 1% Triton X-100), as previously described [47]. The amount of arginase that induced the formation of 1 mmol of urea/min was defined as the unit of enzyme activity.

### 2.10. Multiepitope Peptide Immunization, and Postinfection Humoral Response Assessment

Blood samples were collected from the animals 30 days after immunization and 6 weeks after infection (*n* = 4 animals for the postimmunization assay, *n* = 5 for the postinfection assay). Standard endpoint ELISA was performed (as described elsewhere [42]) to quantify both anti-SLA and anti-peptide antibodies titers (Abs). Briefly, 96-well flat-bottomed microtiter plates (Nunc Immunoplate, Merk, Darmstadt, Germany) were coated for 12 h at 4 °C with 100 μL of SLA (10 μg/mL) or HisDTC (10 μg/mL) and AK peptides (10 μg/mL) diluted in PBS. Serum samples were serially diluted twofold, starting from 1/100. All samples were analyzed individually. As secondary Abs, peroxidase-labeled goat anti-mouse IgG (dilution 1/4000, Southern Biotech, Birmingham, AL, USA) and IgG isotypes (IgG1 and IgG2a, dilution 1/8000, Sigma-Aldrich, USA) were employed. For colorimetric reaction, TMBsubstrate was used. Optical density values were read at 450 nm by using a spectrophotometer (BenchMark Plus, Bio-Rad Laboratories, Hercules, CA, USA). The reciprocal endpoint titer was set as the inverse value of the highest serum dilution factor providing an absorbance three times higher than the negative control.

### 2.11. Statistical Analysis

Data are presented as the mean ± standard deviation (SD) for normally distributed variables and as the median and the interquartile range in the case of the antibody response. The statistical analyses and the graphical representation were conducted using GraphPad Prism software (version 8.3 for Windows, San Diego, CA, USA). For those variables following a normal distribution, we performed the Shapiro–Wilk normality test. Then, analyses were made using one-way ANOVA with the multiple ranges of Tukey’s test. Differences between immunized and control groups are shown with hashes, and differences between groups are shown with asterisks. The antibody response among animals was analyzed using the Mann–Whitney test. Finally, *p*-values ≤ 0.05 were considered to be significant.

## 3. Results

### 3.1. Used of Artificial Neural Network Analysis for Predicting Strongly Binding Epitopes for Human and Mouse MHC Class I and II Molecules

In the present study, to obtain multiepitope peptides capable of strongly binding to human and mouse MHC class I and II molecules, we performed an in silico analysis of six *Leishmania* antigens (four core histones: H2A, H2B, H3, H4; A2 protein, and KMP-11) that had already been suggested to be effectively encoded as DNA vaccines against CL or VL in the murine model [33,42,43].

As there is a wide and diverse genetic human population, we analyzed those proteins for all the human alleles available in the NetMHC database. As mentioned above, HLA (A, B, D, and E) alleles for MHC class I epitopes and HLA (DR, DP and DQ) alleles for MHC class II epitopes were analyzed, as well as H-2 mouse alleles for both MHC class I and II molecules. Hence, 9-mer high binder epitopes for MHC-I were selected for all the proteins. Then, 15-mer high binder epitopes for MHC-II were sought. Subsequently, we focused on six peptides with lengths of 15–24 amino acids (Table 1) that had the greatest number of high-scored binding alleles with MHC class I restricted epitopes, as well as at least one adjacent or overlapping MHC class II-restricted epitope that also scored highly. When possible, sequences were longer to present two MHC-II high-scored binding epitopes. Thus, 6 peptides were selected, that is, one for each protein, as shown in Table 1.

### 3.2. In Silico Analysis of Chimeric Multiepitope Peptides

According to the above data, we designed two multiepitope peptides: HisDTC (TAVLEYLTAELLELSRSLKAINAQMSMSHRTMKIVNSYVEGLRFQSSAIMALQEITRGCVRRMARRGGVK), containing high binding epitopes of the four core histones (H2A, H2B, H3, and H4), and AK (SAEPHKAAVDVGPLSVGPQSVGPLSVGPQAKMHEHSEHFKQKFAELLEQQKA), containing high binding epitopes of the A2 protein and KMP-11.

Both peptides were further analyzed to determine the conservation of potential MHC class I and II epitopes using the programs mentioned before. Both peptides were also submitted to NetTepi. HisDTC was positive for 9 of the 13 different human MHC (HLA) alleles of the common HLA-A and B supertypes, whereas the AK peptide was positive for 6 out of 13. Both chimeric sequences present a high-affinity 9-mer aa epitope for at least one allele of the chimpanzee, macaque, cattle, and pig MHC-I molecules.

### 3.3. Evaluation of the Efficiency of Multiepitope Peptide Immunogenicity in Mice

**Figure 1:**
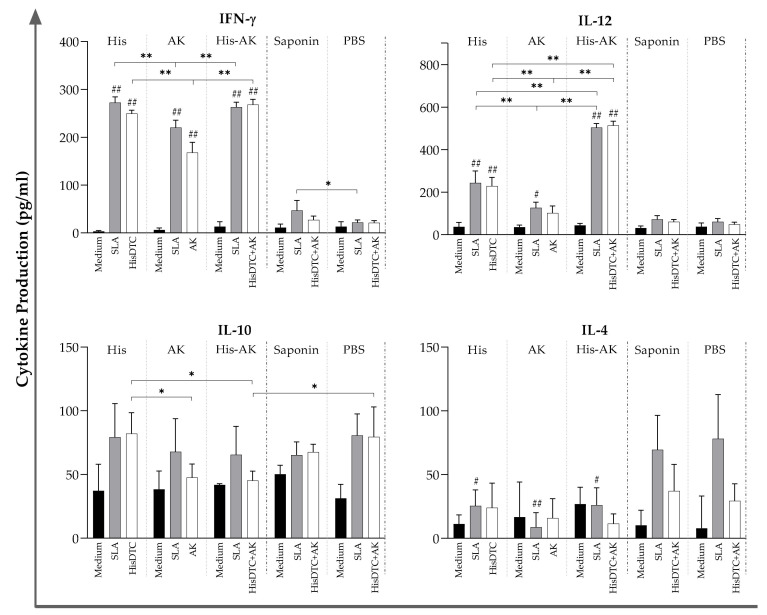
Multiepitope peptide-specific cytokine production of immunized animals prior to infection. Naïve BMDCs stimulated with SLA (soluble *L. infantum* antigen) or the specific peptide used for vaccination were cocultured with spleen cells from immunized animals. ELISA was performed to quantify cytokine production. Data are presented as the mean ± SD. Hash marks indicate significant differences (#, *p* < 0.05; ##, *p* < 0.001) between immunized and control (Saponin and PBS) groups. Asterisks indicate significant differences (*, *p* < 0.05; **, *p* < 0.001).

To evaluate the immunogenicity of HisDTC and AK synthetic multiepitope peptides and the combination of both chimeras (His-AK) generated in BALB/c, we cocultured spleen cells from immunized and control groups with naïve BMDCs. Measurement of cytokine production associated with the Th1 (IFN-γ, IL-12) and Th2 (IL-4, IL-10) profiles was undertaken after stimulation with SLA or peptide used for each immunization group. As shown in Figure 1, splenocytes from immunized groups presented higher production of IFN-γ than control groups, whereas only His and His-AK groups displayed significantly higher levels of IL-12 than Saponin and PBS groups. In addition, increased IL-4 production was only shown by the control groups after SLA or specific peptide stimulation, while no major differences in IL-10 levels were observed between groups or peptides.

**Figure 2:**
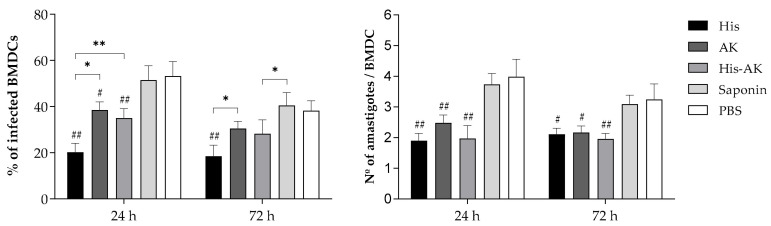
Preinfection assessment of the percentage of infection and the number of amastigotes per infected cell. Naïve BMDCs infected with *L. infantum* were cocultured with splenocytes from immunized animals, and the above parameters were quantified by optical microscopy. Data are depicted as the mean ± SD. Hash marks indicate significant differences (#, *p* < 0.05; ##, *p* < 0.001) between immunized and control (Saponin and PBS) groups. Asterisks indicate significant differences (*, *p* < 0.05; **, *p* < 0.001).

**Figure 3:**
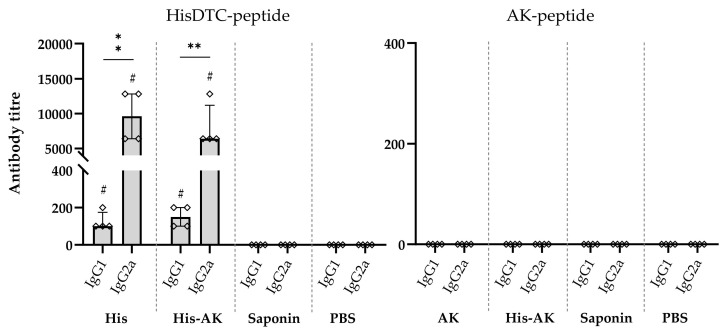
Specific humoral response generated after vaccination. The reciprocal endpoint titer of IgG1 and IgG2a antibodies against HisDTC and AK peptides of each animal was determined (romboids). Data are presented as the median and the interquartile range. Hashes (#) indicate significant differences (*p* < 0.05) between immunized and control (Saponin and PBS) groups. Asterisks indicate significant differences (**, *p* < 0.001).

To further analyze whether the immune response is correlated with an increase in leishmanicidal potential, we evaluated ex vivo parasite killing activity by infecting naïve BMDCs and coculturing splenocytes from immunized mice. The percentage of infected BMDCs and the average number of amastigotes in cocultures from the control groups were higher than those in the immunized groups at 24 and 48 h postinfection (Figure 2). The low infection index, which combines both parameters, indicates a better induction of leishmanicidal activity in BMDCs. Although the three immunized groups presented a significantly lower infection index at 24 and 72 h than the control groups, HisDTC induced a higher killing ability at both time points (Table 2). Finally, to evaluate the multiepitope peptide effect on the humoral response, specific antibodies of IgG class, as well as of IgG1 and IgG2a isotypes, were analyzed in mice immunized with each synthetic peptide, Saponin and PBS, 30 days after the third immunization. According to the results, only HisDTC was able to induce the secretion of peptide-specific IgG antibodies (data not shown), including strongly induced IgG2a and weakly induced production of the IgG1 isotype (Figure 3). Taken together, these results revealed that immunized mice elicited a predominant Th1 cytokine profile together with an IgG subclass redirection to Th1-related IgG2a subclass correlated with an increased leishmanicidal capacity, especially when HisDTC peptide is involved.

### 3.4. Immunization with Multiepitope Peptides Induced a Diminished Parasite Load in Target Organs

**Figure 4:**
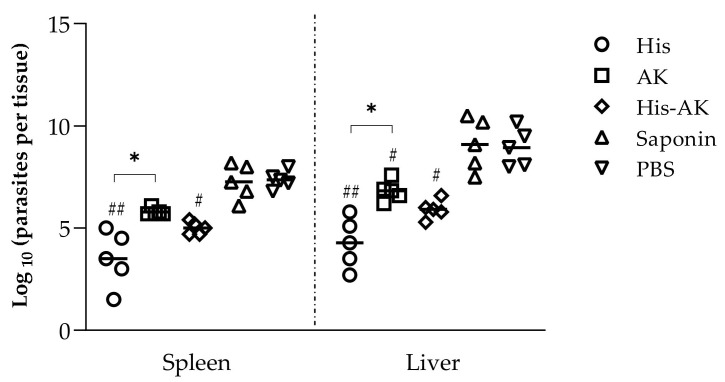
Parasite load in animals after in vivo infection. Six weeks after infection, the animals were sacrificed, and the spleen and liver were subjected to a limiting dilution assay. Data are presented as the mean ± SD. Hash marks indicate significant differences (#, *p* < 0.05; ##, *p* < 0.001) between immunized and control (Saponin and PBS) groups. Asterisks indicate differences (*, *p* < 0.05).

To evaluate whether the immune response elicited by vaccination was maintained during infection and conferred protection, the peptide-specific response was assessed in both vaccinated and control groups infected with *L. infantum* 6 weeks after challenge. His and His-AK groups presented a significant lower parasite burden in both the liver and spleen compared to the Saponin and PBS groups, whereas the AK group only presented a significantly lower amount of parasite in the liver (Figure 4). Moreover, and in keeping with the results found in the preinfection studies in both targeted organs, His-immunized animals presented a significantly (*p* < 0.05) lower parasite burden than the AK group.

### 3.5. Effects of Immunization on the Induction of the Immune Response After L. infantum Challenge

**Figure 5:**
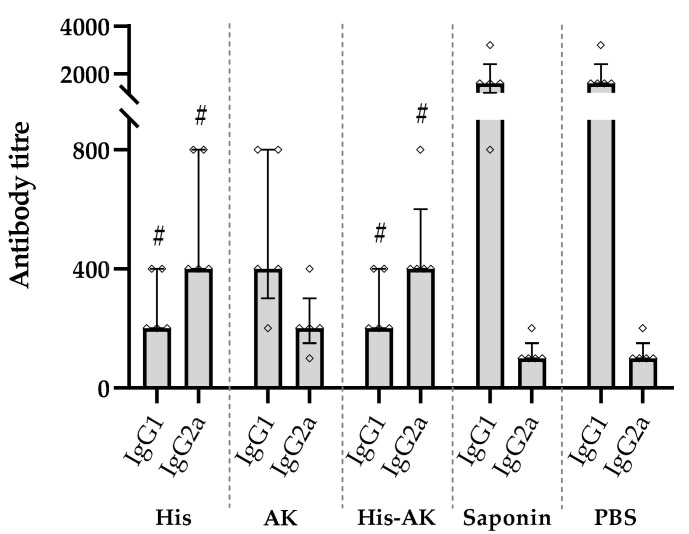
Humoral response after infection. Serum samples were collected from immunized and infected mice. The reactivity against SLA was determined by evaluating the IgG1 and IgG2a isotype antibody levels of each animal (romboids). Data are presented as the median and the interquartile range of the reciprocal endpoint titer. Hashes (#) indicate significant differences (*p* < 0.05) between immunized and control (Saponin and PBS) groups.

After we determined that the vaccination induced a reduction in parasite burden in target organs, especially in those groups immunized with the HisDTC peptide, we analyzed the immune correlates of protection. For that step, we next determined the stimulation of the humoral and cellular responses for SLA and specific peptides. Protection correlated with an IgG subclass redirection to Th1-related IgG2a subclass of SLA-specific antibodies in the His and His-AK mice at 6 weeks after challenge. In contrast, the induction of a predominant humoral response, where IgG production was mainly of the Th2-related IgG1 subclass, could be seen in PBS and Saponin controls (Figure 5).

**Figure 6:**
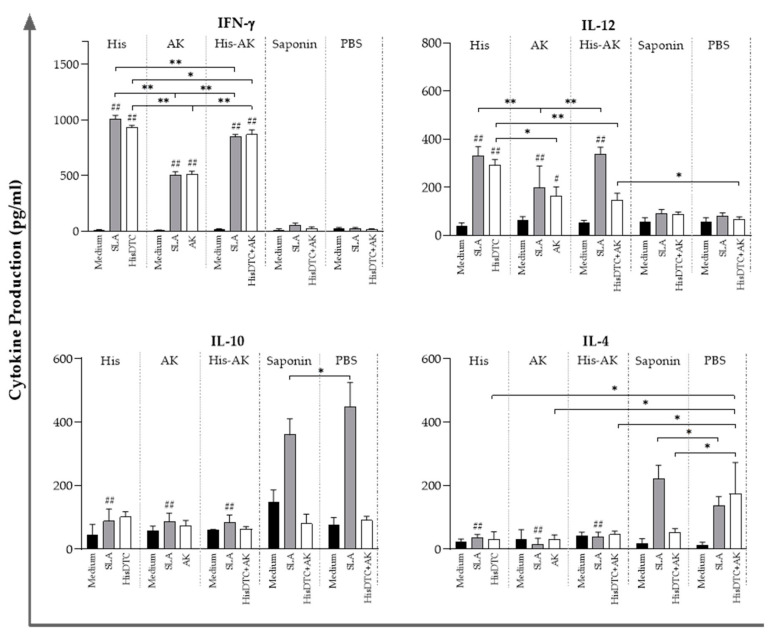
Cytokine production from the supernatant of the coculture system of infected animals. Naïve BMDCs stimulated with SLA (soluble *L. infantum* antigen) or the specific peptide used for vaccination were cocultured with spleen cells from immunized and infected animals. ELISA was performed to quantify cytokine production. Data are presented as the mean ± SD. Hash marks indicate significant differences (#, *p* < 0.05; ##, *p* < 0.001) between immunized and control (Saponin and PBS) groups. Asterisks indicate significant differences (*, *p* < 0.05; **, *p* < 0.001).

The cellular response against SLA and the peptide/s used for each immunization in the *L. infantum*-infected mice were determined by stimulating spleen cells from mice of each group and evaluating cytokine production, as described in Section 3.3. In agreement with the Th1-like profile of the humoral response, an SLA-dependent IFN-γ- and IL-12-predominant response was found in all immunized groups (*p* < 0.001) with respect to the PBS and Saponin groups (Figure 5). Interestingly, when examining the anti-inflammatory Th2 characteristic cytokines IL-10 and IL-4, all immunized groups showed a significant concomitant decrease (*p* < 0.001) compared to the control groups (Figure 6). These results suggested that immunized BALB/c elicited enhanced Th1 characteristic cytokines compared to the Saponin and PBS groups, consistent with the establishment of a protective cellular response. In addition, there were some differences between immunized animals with HisDTC, such as His and His-AK, which presented higher production of proinflammatory cytokines than AK. Although the AK group presented the highest parasite burden among the immunized animals, no differences were found in terms of the anti-inflammatory cytokines IL-10 and IL-4 among them. When splenocytes were stimulated with the specific peptides used for each group immunization, cytokine production followed a similar pattern as with SLA, only slightly less production was observed (Figure 6).

**Figure 7:**
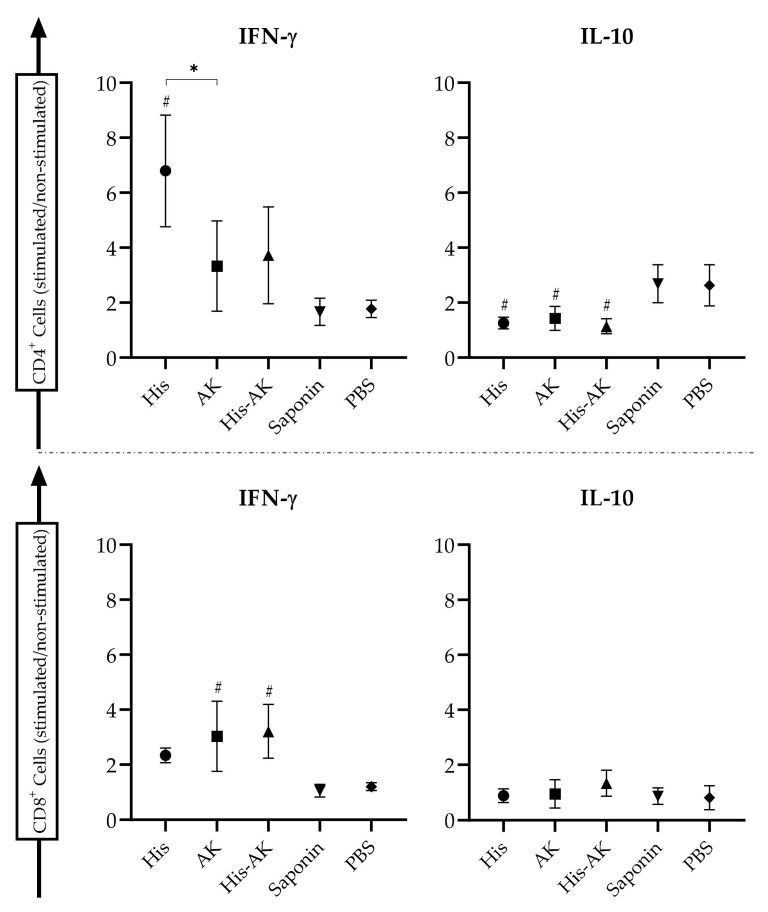
CD4^+^ and CD8^+^ T-cell involvement in the production of IFN-γ and IL-10. Six weeks after the infection, animals were sacrificed, and spleen cell suspensions were stimulated or not with SLA. The intracytoplasmic production of IFN-γ and IL-10 by T-cells was measured by flow cytometry. Data are presented as the ratio between stimulated/nonstimulated cells. Data are presented as the mean ± SD. Hash marks indicate significant differences (#, *p* < 0.05;) between immunized and control (Saponin and PBS) groups. Asterisks indicate significant differences (*, *p* < 0.05).

Furthermore, we explored the percentages of T-cell populations in the spleen from control and vaccinated animals by flow cytometry. SLA-stimulated splenic cell analyses revealed that immunization with multiepitope peptides in combination with saponin induced the production of intracytoplasmic IFN-γ in different ways (Figure 7). The His group was able to stimulate a higher number of IFN-γ-producing CD4^+^ T-cells than the AK, His-AK, and control groups. AK and His-AK showed a higher number of IFN-γ-producing CD8^+^ T-cells than His and control groups. Interestingly, all immunized mice showed a significant concomitant decrease in IL-10-producing CD4^+^ T-cells (Figure 6). Taken together, these data allowed us to conclude that immunization enhances proinflammatory cytokine secretion after experimental infection with *L. infantum*, thereby indicating that the multiepitope peptide-induced IFN-γ production from CD4+ and/or CD8^+^ T-cells are correlated with an IgG2a skewed humoral response, confirming the predictions by in silico analysis.

### 3.6. Multiepitope Immunization Mitigated the Impact of L. Infantum Parasites on Host L-arginine Metabolism

It is well-known that L-arginine in macrophages can be catabolized either by inducible nitric oxide synthase (iNOS) to produce NO or by arginase for polyamine synthesis, which significantly influences the *Leishmania* infection outcome [48,49]. Therefore, we evaluated NO and arginase enzymatic activities in spleens from immunized and infected mice. Immunized groups, especially His, presented higher production of nitrites than the control groups (Table 3). We found that arginase activity was downregulated in the His and His-AK groups in correlation with increased production of nitrite levels. The values for AK were intermediate between HisDTC immunized and Saponin/PBS. Thus, in correlation with the protective immune response profile, HisDTC peptide enhanced the splenocyte ability to induce NO production in response to DC stimulation with SLA in DC-splenocyte coculture.

## 4. Discussion

The increasing incidence of zoonotic VL and other forms of the disease necessitates the development of new control strategies, such as prophylactic or immunomodulatory vaccines. During the last few years, while a considerable number of components have been examined as vaccine candidates across various *Leishmania* species, only a small number have progressed to human or canine clinical trials [50]. A great number of peptide-based vaccines have been tested using individual antigens or a combination of several peptides that are designed as recombinant products by molecular biology [15]. In this context, our group has worked with seven highly immunogenic *Leishmania* antigens (H2A, H2B, H3, H4, A2, KMP11, and HSP70) [27,28,29,30,33]. These antigens have been demonstrated to be able to induce partial protection against *L. infantum*, *L. major* and *L. amazonensis* infection, being encoded as DNA vaccines [33,43]. In addition, we have shown that HisAK70 DNA vaccination followed by an adoptive transfer of BMDCs pulsed with the HisAK70 polyprotein cocktail was successful against an ex vivo *L. infantum* challenge in dogs [34]. In the present study, we attempted to improve the HisAK70 immunization strategy by selecting, through in silico analysis, multiepitope peptides that bind to MHC class I and II molecules.

An effective cellular response against *Leishmania* spp. is essential to control the disease [51]. Therefore, a rationally designed vaccine should have epitopes that are recognized by antigen-presenting cells (APCs) and are able to trigger a rapid T-lymphocyte effector response and maintain long-lasting immune memory, which are critical for protection against the parasite [19,24]. In this context, the discovery of MHC-binding motifs in proteins has enabled the development of algorithms that are able to predict MHC class I- and II-restricted epitopes for presentation to CD8^+^ or CD4^+^ T-cells, respectively [26].

In our study, we performed an in-silico analysis using NetMHC and NetMHCII predicting software to generate two multiepitope peptides: HisDTC from H2A, H2B, H3, and H4 and AK from A2 and KMP11. Among those chimeras, there were several epitopes that presented a binding affinity to a significant number of HLA alleles. When looking for strongly binding epitopes, there are many studies using mouse alleles [24,52] to check whether the epitope selections are able to induce a resistant immune phenotype in the mouse model of VL. Nevertheless, when searching for a possible vaccine candidate for VL, it is important to select not only mouse alleles but also human alleles [23,53,54]. Based on bioinformatics tools, we designed two multiepitope peptides that are able to bind to the greatest number of human and mouse alleles for MHC class I and II molecules. Although NetMHC cannot analyze whether these two sequences present affinity to canine alleles, there appear to be high levels of identity between MHC alleles of dogs and those of humans and mice [55,56].

Furthermore, we analyzed the possibility of those peptides containing promiscuous T-cell epitopes that have the ability to induce T-cell-mediated protective immune responses in other species by binding to several alleles. Both multiepitope peptides presented high-affinity 9-mer amino acid epitopes for at least one allele of the chimpanzee, macaque, cattle, and pig MHC-I molecules. Thus, these promiscuous epitope-driven vaccines may have the capacity to increase the frequency of those who respond in several species, including the dog as the main reservoir for VL in the Mediterranean basin, as previously reported [19].

We corroborated the immunoinformatic approach by screening the immune response generated in mice after immunization with multiepitope peptides by focusing on the main biomarkers of protection, such as proinflammatory cytokines, production of specific immunoglobulins of IgG1/IgG2 isotypes, and CD4^+^ and CD8^+^ T-cell responses. These biomarkers are essential to determine resistance or susceptibility to VL, and they are extensively proven in the literature [19,57]. To that end, we employed a coculture system of spleen cells from the immunized animals and naïve BMDCs previously pulsed with SLA-specific peptides or infected with *L. infantum* metacyclic promastigotes. At 30 days postimmunization, the immunogenicity of HisDTC, AK, and their combination was characterized by IFN-γ and IL-12 production, proinflammatory cytokines with high importance in the activation of Th1 cell responses required for protective immunity against VL. Indeed, high production of IL-4 and IL-10 was only observed in the control groups, indicating the absence of a Th2 response in immunized mice and, therefore, a deficient response to VL.

In keeping with these data, splenocytes from immunized mice were able to induce *Leishmania* killing activity at 24 and 72 h after ex vivo *L. infantum* infection, as reflected by the infection index values (Table 2). Surprisingly, in contrast to previous observations made when used as a DNA vaccine [34,43], when looking for peptide-specific antibody production, HisDTC immunization was able to induce a specific humoral response, IgG2a skewed, whereas AK immunized animals did not. All these data indicate the ability of immunized animals to present a predominantly cellular immune response characterized by the production of Th1 proinflammatory cytokines, with HisDTC being the most efficient.

Since *Leishmania* antigen-specific CD4^+^ Th1 and CD8^+^ T-cells have also been reported as essential requirements of immunity against leishmaniosis [57,58], the role of these populations in the present study was assessed after *L. infantum* challenge of vaccinated mice. Determination of the cytokine profile in splenocytes showed high levels of IL-12 and IFN-γ and low levels of IL-4 and IL-10 in immunized groups, unlike high levels of IL-4 and IL-10 in control groups. In the BALB/c model, several lines of evidence suggest that the control of infection depends not only on the induction of IFN-γ-mediated responses but also on the control of IL-10 and IL-4 cytokines that are associated with pathology [59,60]. The immune response elicited by the His, AK, and His-AK groups conferred a significant reduction in parasite burden, especially in HisDTC-vaccinated mice. Additionally, a correlation between the preinfection Th1 cytokine expression pattern and the reduction of parasites was observed in the immunized groups of mice.

To further investigate the requirements for sustained cellular immunity to *L. infantum*, we analyzed the involvement of CD4^+^ and CD8^+^ T-cells to the production of *Leishmania*-specific IFN-γ and IL-10 in splenocytes from infected mice [61,62]. Immunization with the HisDTC peptide led to the production of IFN-γ by CD4^+^ T cells, whereas immunization with the AK peptide or both together induced the production of IFN-γ by CD8^+^ T-cells. CD8^+^ T-cells, along with CD4^+^ Th1 cells, are involved in the clearance of primary infection through IFN-γ production, which boosts the leishmanicidal capacity of macrophages via nitric oxide (NO) production [15,58]. Only the control groups showed IL-10-producing CD4^+^ T-cells. Indeed, His and His-AK combination groups (probably due to the presence of HisDTC in both formulations), but no AK group, triggered antileishmanial activity based on the upregulation of inducible nitric oxide synthase (NO) and the downregulation of arginase activity after IFN-γ activation. Moreover, the elevated ratio of IgG2a/IgG1 antibodies towards parasites (SLA-specific) in vaccinated protected mice (especially with HisDTC) was consistent with the overall Th1 profile, since previous studies have shown that IFN-γ directly regulates IgG2a class switching [63]. These data demonstrate that the control groups presented a susceptible immune phenotype characterized by an inefficient humoral response leading to disease progression, whereas Th1 cytokines together with the enzyme modulation shown by NO activity and IgG2a bias production induce a resistant immune phenotype that controls disease progression in immunized animals.

Moreover, HisDTC, among both multiepitope peptides, was able to induce the most efficient protective immune response against *L. infantum* infection in mice and was supported by polarized Th1 and/or CD8^+^ T-cell immune responses through antigen presentation in the context of MHC class II and/or MHC class I molecules. The differences between the His and AK groups existed in many parameters, showing that HisDTC has a better capacity of inducing a protective response. Surprisingly, there are few differences between the His and His-AK groups; nevertheless, immunization with the HisDTC peptide seems to be more effective in terms of parasite burden, enzyme modulation, and IFN-γ and IL-12 production. These differences can be due to the amount of peptide, as the His-AK group was immunized with half of the HisDTC peptide compared to the His group. Nevertheless, no synergistic effect of both peptides was obtained, as may be observed with other peptides [53].

Peptide-based vaccination seems to be a good approach to control VL because of its absence of side effects, stability, and reduced cost to scale up. In this context, recently, there have been several studies searching for epitopes using bioinformatics to improve the immune responses generated by some proteins that had already been proven to be efficacious [19,24,64]. These selected epitopes must be able to induce strong, long-lasting cellular immunity. Further research is required to improve multiepitope peptide vaccine protocols based on suitable peptide delivery systems and adjuvant compounds to obtain the desired immune response.

## 5. Conclusions

Our results indicate that bioinformatics is a suitable way to select multiepitope peptides among previously described antigens. Thus, we designed two multiepitope peptides, HisDTC and AK, with high binding affinity MHC class-I- and class-II-restricted epitopes for humans and mice, which are able to promote protective Th1 and CD8^+^ T-cell responses. After an in vivo analysis was performed, HisDTC was determined to be able to induce the best protective immune response in terms of parasite burden, cytokine production, enzyme modulation, and IgG production in both immunized and infected animals. In addition to the use of bioinformatics as a tool for vaccine development, immunogenicity experiments are needed to test the efficacy of in silico analysis-derived peptides, as not all peptides function the same way in the in vivo experiments. The successful protection against VL by using HisDTC contributes to the potential development of synthetic subunit vaccine formulations against multiple *Leishmania* spp. This option may become a promising strategy, not only by promoting protection against leishmaniosis but also by representing a potent therapeutic tool to treat the disease [65]. However, different vaccine delivery systems and adjuvants, such as nanoparticles, should be taken into account in future studies to improve the response, as they can stimulate a long-lasting Th1 immune profile [66,67].

## 6. Patents

The University Complutense of Madrid (Spain) has filed a patent on the HisDTC multiepitope chimera (P202030547).

## Figures and Tables

**Table 1:** Multiepitope peptides derived from the in silico analysis of six *L. infantum* proteins.

		MHC-I				MHC-II			
Protein	Selected Peptide	HLA-A	HLA-B	HLA-C and E	H-2	HLA-DR	HLA-DP	HLA-DQ	H-2
**H2A**	TAVLEYLTAELLELS	13	6	8	1	5	9	15	X
**H2B**	RSLKAINAQMSMSHRTMKIVNSYV	16	12	9	X	16	2	11	4
**H3**	EGLRFQSSAIMALQE	10	3	8	X	17	5	12	3
**H4**	ITRGCVRRMARRGGVK	X	6	X	X	6	1	3	2
**A2**	SAEPHKAAVDVGPLSVGPQSVGPLSVGPQA	X	X	X	4	7	7	8	10
**KMP-11**	KMHE12HSEHFKQKFAELLEQQKA	3	16	5	X	2	7	2	2

*In silico* analyses were performed using NetMHC and NetMHCII software. The numbers of alleles with binding affinity for each locus are represented.

**Table 2:** Infection index calculated by using the percentage of infected cells and the number of amastigotes per infected cell.

Groups:	His	AK	His-AK	Saponin	PBS
**24 h**	3.80 ± 0.43 ^##,a^	9.60 ± 1.40 ^##^	6.94 ± 2.00 ^##^	19.23 ± 2.18	21.17 ± 3.67
**72 h**	3.97 ± 1.28 ^##^	6.59 ± 1.30 ^#^	5.60 ± 1.38 ^##^	12.63 ± 2.62	12.43 ± 2.16

Data are presented as the calculated indexes ± SD. Hashes indicate statistically significant differences (#, *p* < 0.05; ##, *p* < 0.001) between immunized and control (Saponin and PBS) groups. ^a^ indicates significant differences (*p* < 0.05) between the His and AK groups.

**Table 3:** Evaluation of arginase metabolism and nitrite determination in mice infected with *L. infantum.*.

Groups:	mU Arginase Activity	µM Nitrites
**His**	7.70 ± 1.10 ^##,a^	14.53 ± 6.79 ^##,a^
**AK**	20.70 ± 4.40 ^b^	7.57 ± 4.35 ^#^
**His-AK**	12.18 ± 1.81 ^##^	10.77 ± 5.47 ^#^
**Saponin**	29.64 ± 6.32 ^c^	1.67 ± 0.35
**PBS**	38.97 ± 3.10	2.20 ± 1.14

Data are presented as the mean ± SD. Hash marks indicate statistically significant differences (#, *p* < 0.05; ##, *p* < 0.001) between immunized and control (Saponin and PBS) groups. ^a^ indicates significant differences (*p* < 0.05) with the AK group. ^b^ indicates significant differences (*p* < 0.001) with the PBS group. ^c^ indicates significant differences (*p* < 0.05) with the PBS group.
